# Amygdalar activity measured using FDG-PET/CT at head and neck cancer staging independently predicts survival

**DOI:** 10.1371/journal.pone.0279235

**Published:** 2023-08-04

**Authors:** Malek Z. O. Hassan, Ahmed Tawakol, Ying Wang, Raza M. Alvi, Magid Awadalla, Maeve Jones-O’Connor, Rula B. Bakar, Dahlia Banerji, Adam Rokicki, Lili Zhang, Connor P. Mulligan, Michael T. Osborne, Azmaeen Zarif, Basma Hammad, Annie W. Chan, Lori J. Wirth, Erica T. Warner, Roger K. Pitman, Katrina A. Armstrong, Daniel Addison, Tomas G. Neilan

**Affiliations:** 1 Cardiovascular Imaging Research Center, Department of Radiology and Division of Cardiology, Massachusetts General Hospital, Harvard Medical School, Boston, Massachusetts, United States of America; 2 Royal Papworth Hospital, Trumpington, Cambridge, United Kingdom; 3 Cardio-Oncology Program, Division of Cardiology, Department of Medicine, Massachusetts General Hospital, Harvard Medical School, Boston, Massachusetts, United States of America; 4 Nuclear Cardiology, Division of Cardiology, Department of Medicine, Massachusetts General Hospital, Harvard Medical School, Boston, Massachusetts, United States of America; 5 Department of Nuclear Medicine, The First Hospital of China Medical University, Shenyang, China; 6 Department of Medical Sciences, Oxford University, Oxford, United Kingdom; 7 Department of Radiation Oncology, Massachusetts General Hospital, Harvard Medical School, Boston, Massachusetts, United States of America; 8 Division of Oncology, Department of Medicine, Massachusetts General Hospital, Harvard Medical School, Boston, Massachusetts, United States of America; 9 Clinical Translational Epidemiology Unit, Department of Medicine, Massachusetts General Hospital, Boston, Massachusetts, United States of America; 10 Department of Psychiatry, Massachusetts General Hospital, Charlestown, Massachusetts, United States of America; 11 Department of Medicine, Massachusetts General Hospital and Harvard Medical School, Boston, Massachusetts, United States of America; 12 Division of Cardiology, Department of Medicine, Ohio State University, Columbus, Ohio, United States of America; University of Kurdistan Hewler, IRAQ

## Abstract

**Importance:**

The mechanisms underlying the association between chronic stress and higher mortality among individuals with cancer remain incompletely understood.

**Objective:**

To test the hypotheses that among individuals with active head and neck cancer, that higher stress-associated neural activity (ie. metabolic amygdalar activity [AmygA]) at cancer staging associates with survival.

**Design:**

Retrospective cohort study.

**Setting:**

Academic Medical Center (Massachusetts General Hospital, Boston).

**Participants:**

240 patients with head and neck cancer (HNCA) who underwent ^18^F-FDG-PET/CT imaging as part of initial cancer staging.

**Measurements:**

^18^F-FDG uptake in the amygdala was determined by placing circular regions of interest in the right and left amygdalae and measuring the mean tracer accumulation (i.e., standardized uptake value [SUV]) in each region of interest. Amygdalar uptake was corrected for background cerebral activity (mean temporal lobe SUV).

**Results:**

Among individuals with HNCA (age 59±13 years; 30% female), 67 died over a median follow-up period of 3 years (IQR: 1.7–5.1). AmygA associated with heightened bone marrow activity, leukocytosis, and C-reactive protein (P<0.05 each). In adjusted and unadjusted analyses, AmygA associated with subsequent mortality (HR [95% CI]: 1.35, [1.07–1.70], P = 0.009); the association persisted in stratified subset analyses restricted to patients with advanced cancer stage (P<0.001). Individuals within the highest tertile of AmygA experienced a 2-fold higher mortality rate compared to others (P = 0.01). The median progression-free survival was 25 months in patients with higher AmygA (upper tertile) as compared with 36.5 months in other individuals (HR for progression or death [95%CI], 1.83 [1.24–2.68], P = 0.001).

**Conclusions and relevance:**

AmygA, quantified on routine ^18^F-FDG-PET/CT images obtained at cancer staging, independently and robustly predicts mortality and cancer progression among patients with HNCA. Future studies should test whether strategies that attenuate AmygA (or its downstream biological consequences) may improve cancer survival.

## Introduction

Cancer is one of the leading causes of death in the developed world [1]. Multiple lines of evidence demonstrate that chronic psychological stress associates with poorer cancer outcomes [2–4]. In animal models, stress activates the immune system, leading to an increased production of pro-inflammatory cytokines [5], redistribution of immune cell populations [6–8]. Together, these changes appear to accelerate tumor growth, and metastases [9–11]. However, in humans, the mechanism linking stress to poorer cancer outcomes remains incompletely defined. Accordingly, a better understanding of the mechanism linking stress to adverse cancer outcomes in humans is needed.

Advanced imaging methods have greatly facilitated the evaluation of the pathological mechanisms linking stress to human diseases [12, 13]. External stressors activate the brain’s salience network, a group of interconnected structures within which the amygdala, a limbic structure, plays a critical role [14]. The amygdala’s resting metabolic activity (AmygA) can be quantified using ^18^F-fluorodeoxyglucose positron emission tomography/computed tomography (^18^F-FDG-PET/CT), providing a physiologic measure that associates with anxious temperament in animal models [15] and perceived stress in humans [16], and is heightened in conditions of chronic stress [17, 18]. We recently studied the relationship between AmygA and cardiovascular events in an cohort of 293 individuals without active malignancy or known cardiovascular disease (CVD) who underwent a clinical ^18^F-FDG-PET/CT. In that study, higher AmygA independently associated with an increased risk of subsequent incident CVD events. Further, mediation analysis suggested that the link between stress and CVD may include a serial pathway of: ↑stress → ↑AmygA → ↑hematopoietic tissue activity → ↑arterial inflammation → ↑CVD risk [16, 19].

Given the well-describe association between stress and cancer risk, we hypothesized that heightened stress-associated neurobiological activity (e.g. AmygA) may likewise associate with an increased risk of cancer-related mortality. Accordingly, herein we tested the hypotheses among in 240 individuals with a homogenous cancer type, viz., head and neck cancer (HNCA), that AmygA measured during staging via ^18^F-FDG-PET/CT independently predicts cancer progression and survival.

## Methods

### Study design and participants

**Fig 1:**
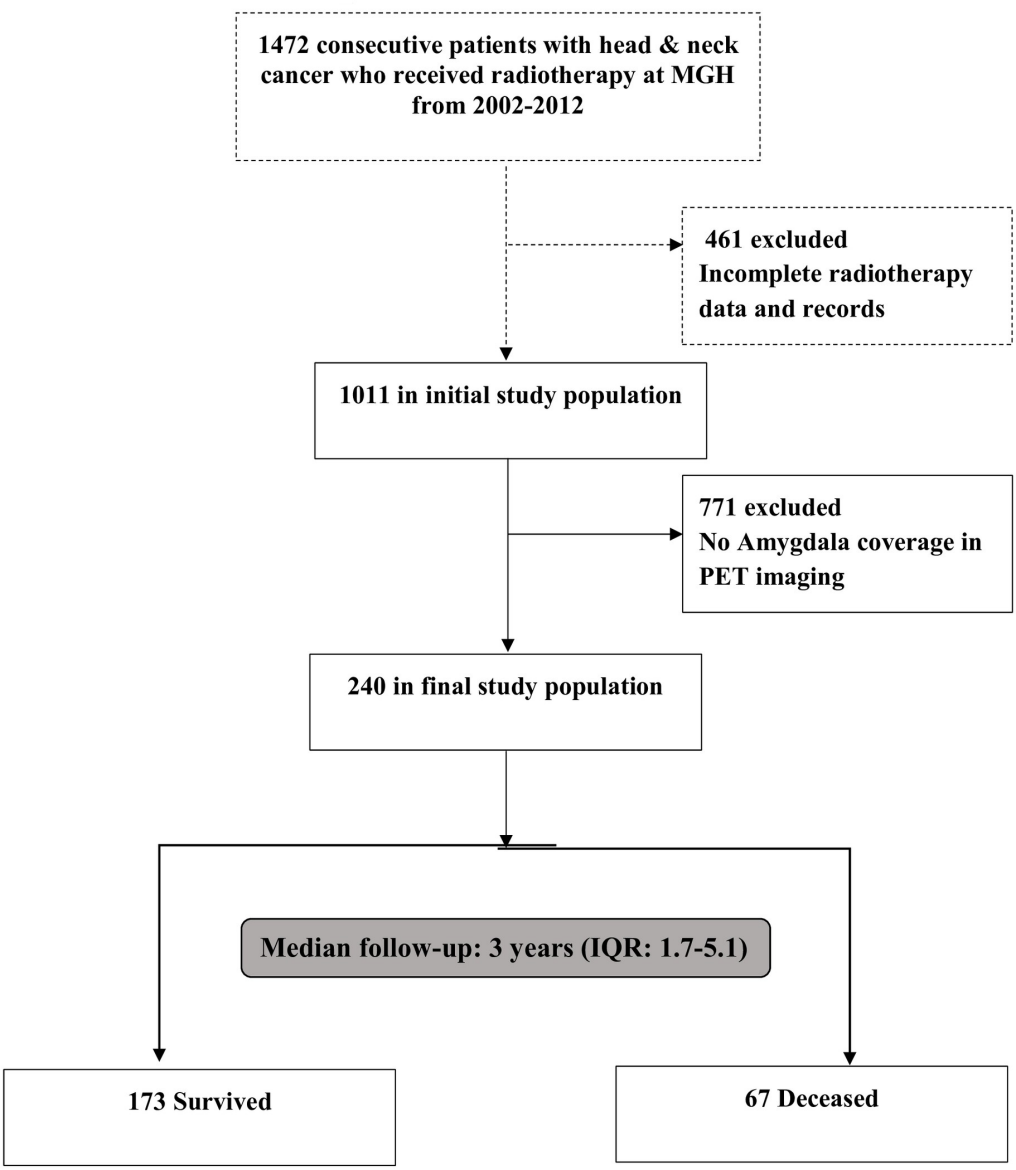
Study consort diagram. From a registry of all patients with head and neck cancer staged at a single academic center over a 10-year period, those with amygdala in the ^18^F-FDG-PET/CT imaging field were included.

From an institutional database at the Massachusetts General Hospital (Boston, MA, USA), we retrospectively identified all consecutive patients with HNCA (all pathological subtypes were included) over 11-years from January 2002 to December 2012 (Fig 1). The database was initially derived to characterize the link between radiation therapy for HNCA and carotid artery disease [20, 21] and thus includes only those with HNCA who underwent radiation therapy. Among those patients, we included all individuals who underwent ^18^F-FDG-PET/CT for cancer staging prior to cancer treatment initiation in whom the amygdala was included in the imaging field of view. The Human Subjects Research Review Committee of our institution approved the study protocol (#2014P001394) and waived informed consent.

### Study variables

Data collection, including CVD and cancer-specific variables and death adjudication were performed manually by two teams of independent investigators. The image analysts were blinded to all subject identifiers and clinical data; clinical analysts were blinded to imaging data. Covariates of interest included age, sex, body mass index (BMI), CVD risk factors, CVD medications and atherosclerotic CVD risk score (ASCVD; a marker of overall CVD risk). Cancer-specific variables included Eastern Cooperative Oncology Group (ECOG) performance status, lymph node involvement, surgical treatment, radiation dose, chemotherapy use, stage, and type of HNCA, as previously defined [20, 21]. Laboratory testing variables included serum sodium, creatinine, white cell count, and hematocrit, recorded from the electronic health record during cancer treatment.

### Procedures

^18^F-FDG was given intravenously at a dose of ~370 MBq after a six hours fast. After tracer injection, individuals sat in a quiet waiting room; imaging was performed approximately one hour later using a PET/CT scanner (Biograph 64, Siemens Healthcare, Erlangen, Germany or similar). A non-gated, non-contrast-enhanced CT (120 keV, ~50 mAs) was obtained for attenuation correction. Analysis of amygdalar activity (AmygA) was performed by a radiologist (YW) who was blinded to all other clinical data using previously described methods [16, 22]. In brief, ^18^F-FDG uptake in the amygdala was determined by placing circular regions of interest in the right and left amygdalae and measuring the mean tracer accumulation (i.e., standardized uptake value [SUV]) in each region of interest. Amygdalar uptake was corrected for background cerebral activity (mean temporal lobe SUV). Bone marrow activity was also measured to provide assessments of leukopoietic activity, according to previously validated methods [16, 22, 23]. To derive this measurement, the mean SUVs were derived by placing a region of interest over axial sections of individual vertebrae from L1 to L5; target-to-background ratios (TBRs) were calculated by dividing the target tissue SUVs by venous blood background activity.

### Outcomes

The primary outcome of interest was all-cause mortality. Death was determined through Social Security death index (SSDI) and confirmed by a board-certified physician blinded to ^18^F-FDG-PET/CT data (DA) using individual electronic health record review. The secondary outcome of interest was progression-free survival which was defined as the time from PET/CT imaging to the first detection of cancer progression or death from cancer, whichever occurred first. Cancer progression was measured using standard RECIST criteria [24].

### Statistical analysis

Continuous data are presented as mean±SD. Comparisons between groups (survived versus deceased) were performed with the use of an independent sample t-test for continuous variables, Fisher’s exact test for categorical variables, and the Wilcoxon rank-sum test for ordinal variables. Pearson product-moment correlation was used to assess univariate associations for normally distributed variables, and Spearman correlation coefficients for non-normally distributed variables. Hazard ratios (HR) for the association of AmygA with events were estimated using Cox proportional hazard models with follow-up time used as the time scale. HRs were assessed with and without the addition of potential confounders as covariates, and 95% confidence intervals (CIs) were estimated for each standard deviation increase in AmygA. We performed log-rank tests to generate Kaplan-Meier estimates and associated curves of survival, comparing mortality in patients with high (upper tertile) vs. low (lower two tertile) AmygA. For robustness, we used both median values and Youden index [25, 26], as alternate thresholds for high AmygA. To additionally test the robustness of our findings, we conducted multiple sensitivity and pre-specified sub-group analyses. Specifically, primary subgroups analyses among all patients were stratified by age (≤65 years vs. >65 years), sex, and those with advance disease were stratified by age, sex, BMI category, and ECOG performance status (≤1 vs >1). For advanced disease analysis, cancer stage I-II was categorized as localized/regional and stage III-IV as advanced/distant. We also tested whether loss to follow-up or disease stage influenced our results by restricting the analyses to patients with no prior history of cancer, those with no history of CVD events, uncensored patients, and patients with advance disease. Statistical significance was determined as two-tailed P-value of < 0.05. P-values for interaction analyses and multiple comparisons were adjusted using Bonferroni method. All statistical analyses were performed with the use of R, Version 1.0.143 (R Foundation for Statistical Computing).

## Results

### Baseline characteristics

Baseline characteristics for the final study cohort of 240 individuals appear in Table 1. Mean age was 59±13 years (range 10–89 years); 30% were women (Table 2). Overall, 11% had diabetes, 47% had hypertension, 66% were active or prior cigarette smokers, and 14% had a diagnosis of depression. Oropharyngeal carcinoma was the most common cancer type. When comparing the 240 individuals who were part of the final study cohort compared to those who were excluded due to lack of amygdalar imaging, excluded patients were more likely to have a higher cancer stage and ECOG status at presentation (S1 andS2 Tables).

**Table 1:** Characteristics and laboratory values of participants at time of cancer staging.

Variable	All (240)	Survived (173)	Deceased (67)	P-value
**Age (yrs)**	**59 (13)**	**59 (13)**	**61 (15)**	**0.26**
**Female sex, n (%)**	**73 (30)**	**52 (30)**	**21 (31)**	**0.97**
**Body Mass Index (kg/m^2^)**	**27.3 (5.7)**	**27.4 (5.7)**	**27.1 (5.8)**	**0.70**
**Psychiatric History, n (%)**				
**Depression**	**33 (13)**	**23 (13)**	**10 (14)**	**0.64**
**Anti-depressant medication**	**29 (12)**	**19 (11)**	**10 (15)**	**0.54**
**Anti-anxiety medication**	**48 (20)**	**37 (21)**	**11 (16)**	**0.49**
**Cardiovascular risk factors, n (%)**				
**Diabetes**	**26 (10)**	**17 (9)**	**9 (13)**	**0.57**
**Hypertension**	**113 (47)**	**79 (45)**	**34 (50)**	**0.57**
**Dyslipidemia**	**64 (26)**	**43 (24)**	**21 (31)**	**0.39**
**Smoking**	**158 (65)**	**107 (61)**	**51 (76)**	**0.05**
**Mean ASCVD 10-year risk**	**12 (14)**	**12 (13)**	**15 (16)**	**0.18**
**Heart failure**	**7 (2)**	**4 (2)**	**3 (5)**	**0.93**
**Ischemic heart disease**	**20 (8)**	**16 (9)**	**4 (6)**	**1**
**Myocardial infarction**	**14 (5)**	**8 (5)**	**6 (9)**	**0.57**
**Stroke**	**8 (3)**	**6 (4)**	**2 (3)**	**0.33**
**Laboratory Values**				
**Hematocrit**	**38 (5)**	**38 (5)**	**37 (5)**	**0.38**
**Total Cholesterol (mg/dL)**	**172 (28)**	**173 (28)**	**168 (27)**	**0.91**
**LDL (mg/dL)**	**96 (26)**	**98 (26)**	**92 (27)**	**0.21**
**HDL (mg/dL)**	**52 (13)**	**52 (14)**	**53 (12)**	**0.12**
**Triglycerides (mg/dL)**	**166 (72)**	**162 (70)**	**176 (77)**	**0.91**
**Glucose (mg/dL)**	**112 (39)**	**112 (35)**	**114 (47)**	**0.7**
**HbA1C (%)**	**6 (1.1)**	**5.9 (1)**	**6.3 (1.3)**	**0.28**
**Sodium (mg/dL)**	**138 (3)**	**138 (3)**	**137 (3)**	**0.46**
**Creatinine (mg/dL)**	**0.93 (0.3)**	**0.92 (0.2)**	**0.96 (0.5)**	**0.44**
**Cardiovascular medications, n (%)**				
**Statins**	**67 (28)**	**47 (27)**	**20 (30)**	**0.90**
**Beta-blockers**	**59 (25)**	**41 (24)**	**18 (27)**	**0.8**
**Aspirin**	**55 (23)**	**39 (23)**	**16 (24)**	**0.73**
**Angiotensin-converting enzyme inhibitor**	**44 (18)**	**32 (18)**	**12 (17)**	**0.96**
**Angiotensin-receptor blockers**	**16 (7)**	**11 (6)**	**5 (8)**	**1**
**Calcium channel blockers**	**17 (7)**	**9 (5)**	**8 (12)**	**0.99**
**Coumadin**	**9 (4)**	**5 (3)**	**4 (6)**	**0.12**

**Table 2:** Cancer characteristics and treatments of participants.

Variable	All (240)	Survived (173)	Deceased (67)	P-value
**Type of Head and Neck Cancer, n (%)**				
**Laryngeal**	**29 (12)**	**21 (12)**	**8 (11)**	**1**
**Oropharyngeal**	**94 (39)**	**76 (43)**	**18 (26)**	**0.02**
**Hypopharyngeal**	**12 (5)**	**8 (4)**	**4 (6)**	**0.92**
**Nasopharyngeal**	**21 (8)**	**14 (8)**	**7 (10)**	**0.75**
**Other**	**93 (39)**	**58 (34)**	**35 (50)**	**0.032**
**Cancer Stage, n (%)**				**0.13**
**Stage Ι**	**7 (2)**	**4 (2)**	**3 (4)**	
**Stage ΙΙ**	**69 (28)**	**56 (32)**	**13 (19)**	
**Stage ΙΙΙ**	**48 (20)**	**35 (20)**	**12 (18)**	
**Stage IV**	**116 (48)**	**77 (44)**	**39 (58)**	
**Detectable Lymph nodes**	**198 (82)**	**140 (80)**	**58 (87)**	**0.44**
**Distant Metastases**	**72 (30)**	**45 (26)**	**27 (40)**	**0.05**
**ECOG status, n (%)**				**0.64**
**0**	**126 (52)**	**93 (53)**	**33 (49)**	
**1**	**74 (30)**	**54 (31)**	**20 (30)**	
**2**	**36 (15)**	**24 (13)**	**12 (18)**	
**3**	**4 (2)**	**2 (1)**	**2 (3)**	
**Radiation characterstics**				
**Mean radiation dose (mSv)**	**66.36 (20.82)**	**67.45 (19.60)**	**63.72 (23.45)**	**0.21**
**Proton**	**17 (7)**	**11 (7)**	**6 (9)**	**0.76**
**Chemotherapy**	**186 (78)**	**133 (77)**	**53 (79)**	**0.86**
**Type of chemotherapy, n (%)**				
**Anthracycline**	**14 (6)**	**9 (5)**	**5 (8)**	**0.72**
**Taxol**	**94 (39)**	**69 (40)**	**25 (37)**	**0.83**
**5 FU**	**25 (10)**	**14 (8)**	**11 (16)**	**0.09**
**Platinum**	**154 (64)**	**111 (64)**	**43 (64)**	**1**
**Other**	**67 (28)**	**46 (26)**	**21 (31)**	**0.56**

There were 67 deaths over a median follow-up period of 3 years (IQR: 1.7–5.1). Of these, 60 deaths resulted from cancer progression, three from infection, three from major bleeding, and one from trauma. Both cardiovascular risk (ASCVD score) and cancer stage were higher among those who died; otherwise, there were no major differences in cancer or non-cancer related characteristics between those who died and those who survived (Tables 1 and 2). Similar results were noticed when comparing patients who progressed and patients who did not progress (S3 and S4 Tables).

### Associations between amygdalar activity, clinical variables, and inflammation

**Fig 2:**
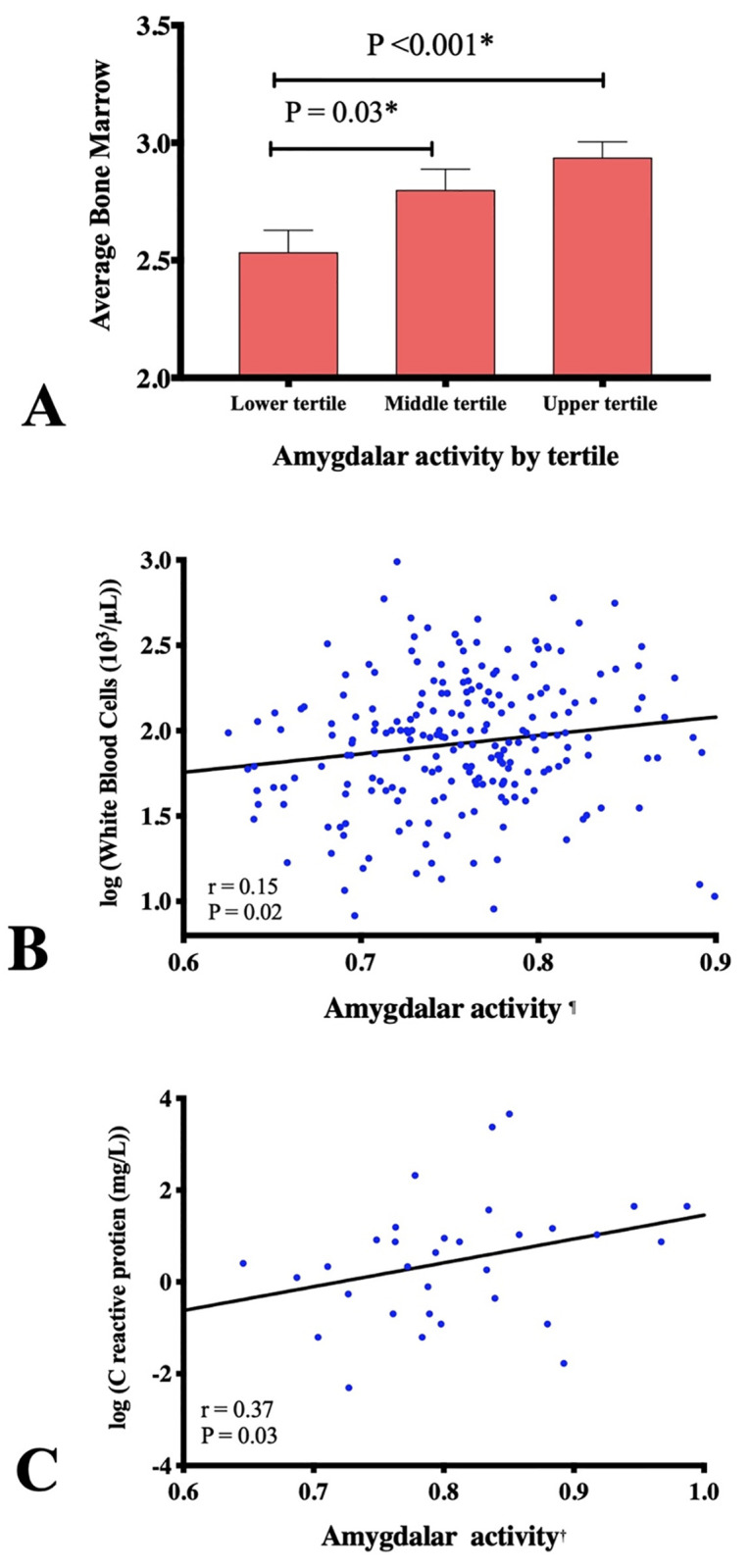
Tissue and biomarker associations with amygdalar activity. Association between tertiles amygdala activity at cancer staging and bone marrow activity (A), white blood cell count (B) and C-reactive protein (C).

Associations between AmygA and clinical variables appear in S1 Table. In brief, higher AmygA associated with higher cancer stage and higher ECOG status. AmygA also correlated with bone marrow activity of hematopoietic activity (r = 0.28, P <0.001), where individuals with lower AmygA (lowest tertile) had lower bone marrow activity compared to those with higher AmygA (Fig 2A). Similarly, AmygA associated with circulating measures of inflammation, including: white blood cell count (r = 0.16, P <0.01, Fig 2A), and C-reactive protein (r = 0.37, P = 0.03, Fig 2B). Furthermore, AmygA was inversely associated with hematocrit in men (r = -0.26, P = 0.03) and in women (r = -0.15, P = 0.02).

### Amygdalar activity vs. outcomes

**Fig 3:**
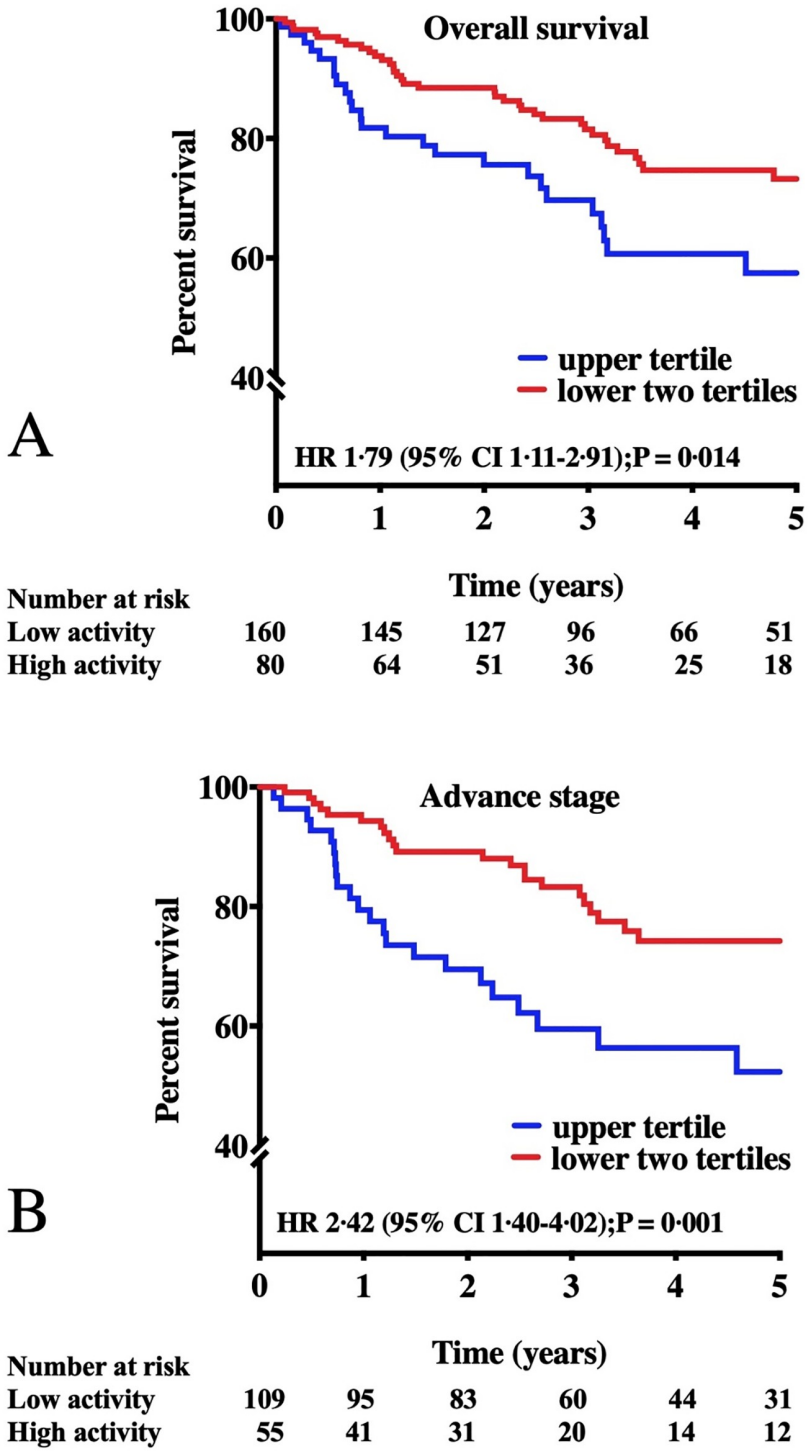
Amygdalar activity vs survival. Kaplan-Meier survival curves of low vs. high amygdalar activity based on the upper tertile vs. the lower two tertiles for all patients (A) and among those with advanced disease only (B) are presented. Amygdalar activity is measured as the mean activity of both amygdalae corrected for background cerebral tissue activity. The P-values were calculated using the log-rank test; Cox regression analyses were used to calculate hazard ratios.

**Fig 4:**
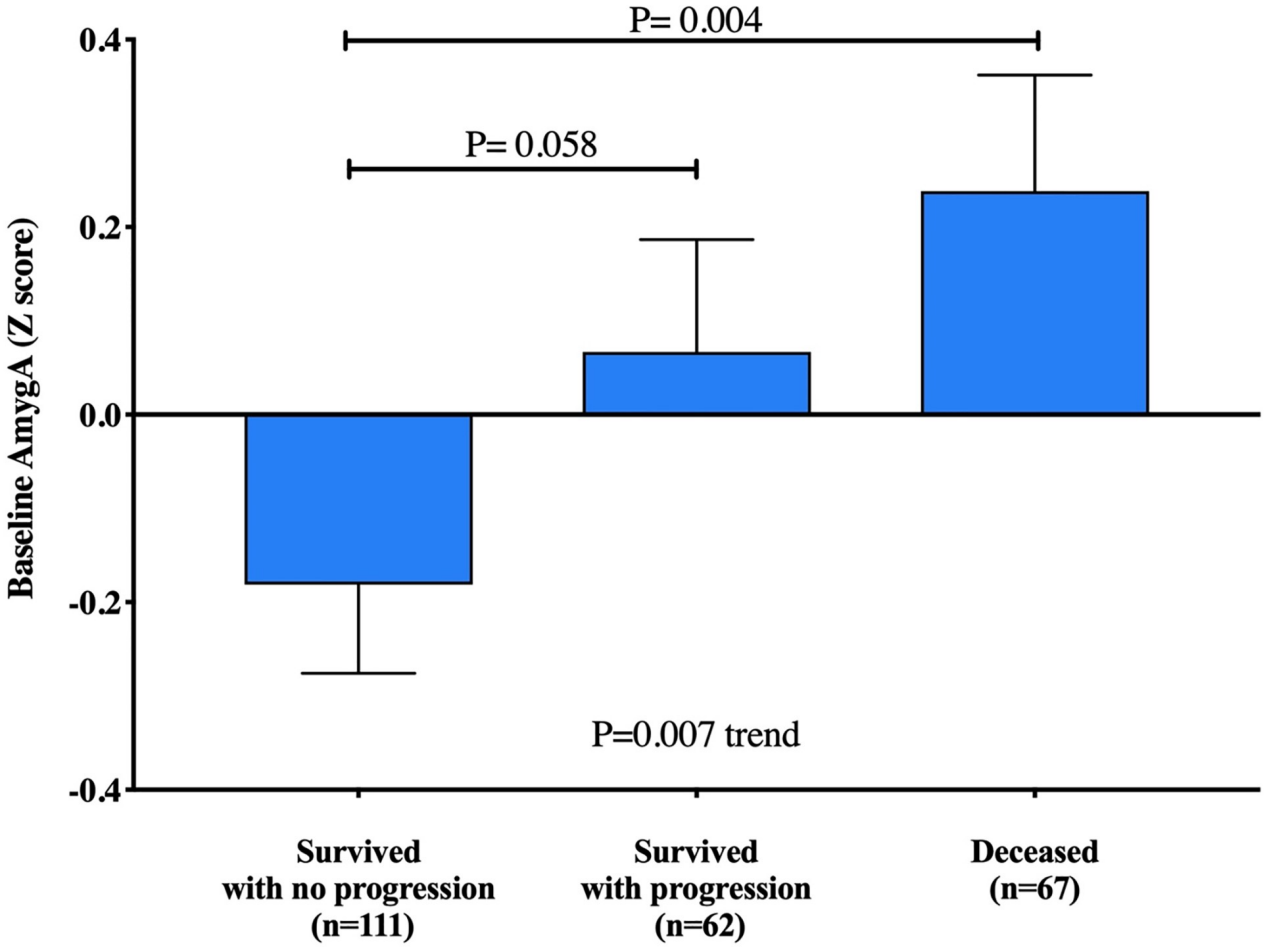
AmygA vs. progression-free survival. A pairwise comparison of adjusted amygdalar activity (AmygA z-score) between: A) individuals who survived without cancer progression, B) individuals who survived with evidence of cancer progression, and C) individuals who died during follow-up.

**Fig 5:**
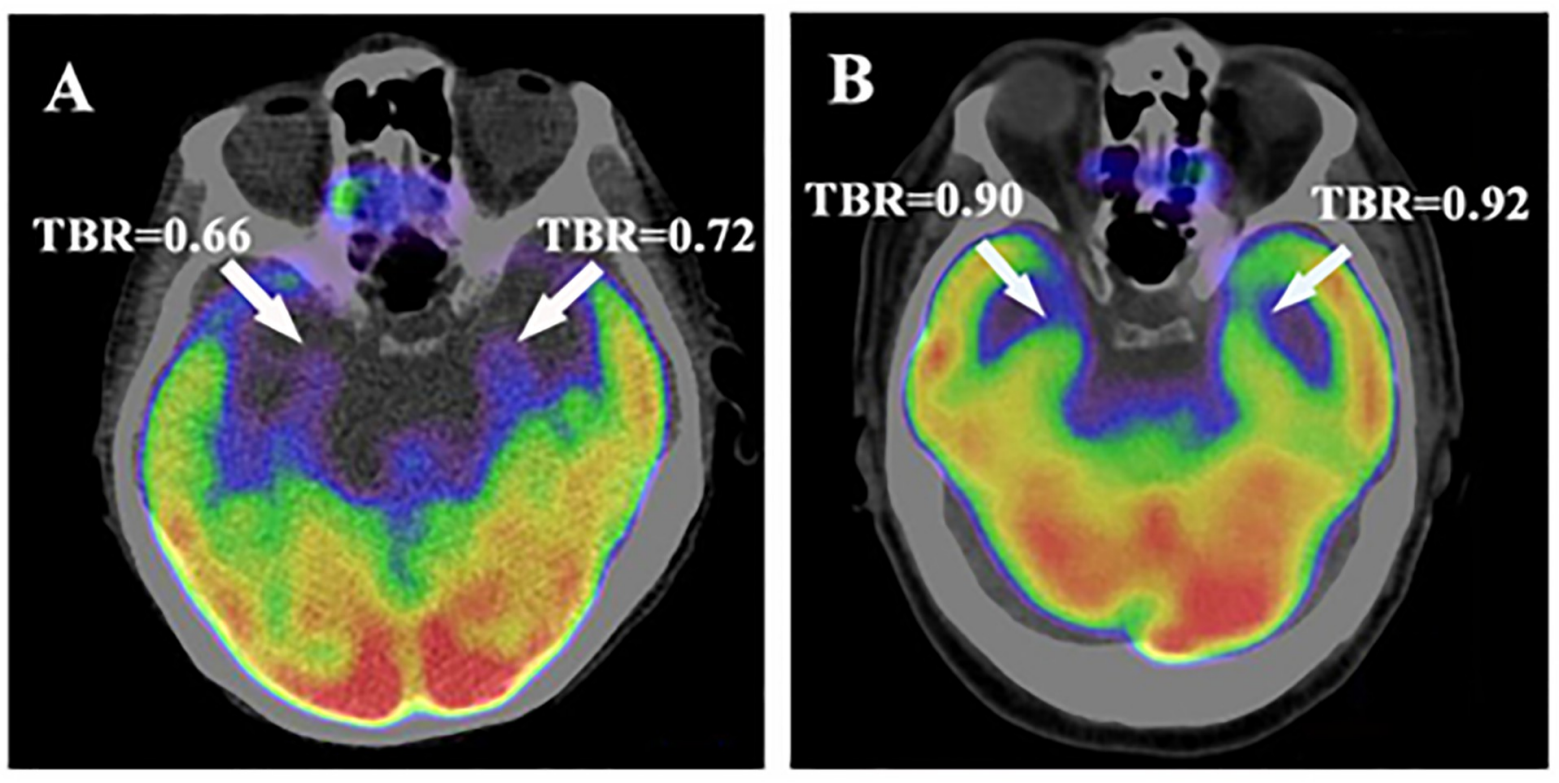
Representative images. Axial views of the amygdala. Higher amygdalar uptake of ^18^F-FDG in a patient who died (A) compared to a patient who survived (B) after a diagnosis of head and neck cancer. These images were recorded at the initial cancer staging prior to cancer treatment. Both individuals had the same stage and type of cancer.

AmygA at staging strongly predicted subsequent mortality (Table 3). Each standard deviation increase in AmygA was associated with a 35% increased risk of death (HR [95% CI]: 1.35, [1.07–1.70], P = 0.009). The associations between AmygA and survival remained significant after adjustment for age, sex, CVD risk factors, cancer-related mortality risk factors, and baseline psychiatric history (Table 3). When we dichotomized amygdalar activity as “high” vs “low” (using the upper tertile threshold), we observed an approximately two-fold higher mortality among those with a higher AmygA (Fig 3A, S6 Table). Alternate thresholds for high AmygA yielded similarly robust results (S7 Table, S1 and S2 Figs). In sensitivity analyses, the relationship between AmygA and survival remained robust when the analyses were limited to patients with no loss to follow-up during the study period (uncensored patients), with no history of prior malignancy, with no history of a CVD event, adult over 40 years of age and with advanced disease (S8). The excess mortality risk associated with high AmygA remained significant among the subgroup of patients with advanced cancer stage at baseline (Fig 3B). We furthermore assessed the relationship between baseline AmygA and progression-free survival. The median time to cancer progression or cancer death was 25 months in patients with higher AmygA (highest tertile) as compared with 36.5 months in individuals with lower AmygA (HR [95%CI], 1.83 [1.24–2.68], P = 0.001, S3 Fig). Additionally, we observed a graded increase in AmygA across individuals grouped by cancer progression. Individuals who had no evidence of disease progression had the lowest AmygA, those who died during follow-up had highest AmygA, and survivors with progression had intermediate baseline AmygA (P = 0.007 for trend, Fig 4). This trend remained significant after adjusting for age, gender, and cancer stage (P = 0.029). Representative images of amygdalar uptake of ^18^F-FDG recorded at the initial cancer of staging are shown in Fig 5.

**Table 3:** Unadjusted and adjusted analyses of amygdalar activity vs. outcomes.

Covariates	Risk of Death HR [95% CI]	P-value
**Unadjusted**	**1.35 [1.07–1.70]**	**0.009**
**Age and sex**	**1.34 [1.06–1.69]**	**0.013**
**Combined cardiac risk factors** ^¶^	**1.29 [1.03–1.62]**	**0.027**
**Combined cancer risk factors** ^†^	**1.32 [1.03–1.69]**	**0.025**
**Combined Psychiatric risk factors** ^¥^	**1.35 [1.07–1.70]**	**0.009**

AmygA activity was quantified as mean bilateral amygdalar activity corrected for background cerebral activity. The association between AmygA was corrected for accepted survival factors which were entered as cofactors in a stepwise manner.

^¶^ The model was adjusted for age, sex, ASCVD risk score, prevalent diabetes, prevalent hypertension, prevalent dyslipidemia, and history of prior major CVD events at baseline.

^†^ The model was adjusted for age, sex, concurrent chemotherapy, surgery, radiation dose, hematocrit, and metastatic disease at baseline.

^¥^The model was adjusted for age, sex, marital status, and history of depression or anxiety at baseline.

Numbers in parentheses are 95% CIs. Abbreviations: AmygA-amygdalar activity; ASCVD-Atherosclerotic cardiovascular disease; CI-confidence interval.

## Discussion

We observed, for the first time in humans, that stress-associated neurobiological activity (measured as amygdalar activity, [AmygA]) at staging on routine FDG-PET/CT scans predicts mortality and progression-free survival among patients with head and neck cancer. The associations between AmygA and outcomes were independent of CVD risk, cancer stage, and cancer therapy, and remained robust when analyses were limited to men, women, or individuals with advanced disease. Moreover, the study points to a plausible biological mechanism (through stress-associated neural pathway activation resulting in higher inflammation and oncologic disease progression), which may represent a target for therapeutic modulation.

The mechanisms through which AmygA associates with increased cancer mortality in HNCA are incompletely understood; however, this study provides some plausible hypotheses. The amygdala is a highly-conserved brain region located within the temporal lobe [27, 28] that plays a key role in emotional regulation. Stress exposure has been found to increases excitability and activity of the amygdala, leading to heightened release of neurotransmitters (e.g., dopamine, noradrenaline, serotonin) in response to stress [29–32]. These neurotransmitters have been shown to exert adverse effects on both vascular and cancer biology and can directly modulate several key processes related to tumor progression and angiogenesis [33–36]. For example, noradrenaline can increase cancer cell survival and tumor angiogenesis through activation of catecholamine-sensitive protein kinases [2]. Dopamine results in heightened bone marrow activity and a resultant increase in tumor angiogenesis [37] Additionally, the amygdala’s axonal projections to the brainstem play an important role in the sympathetic responses to stress [38]. Animal studies have shown that brainstem-derived sympathetic efferents, when activated by stress, lead to increased bone marrow hematopoietic stem and progenitor cell proliferation in addition to accelerated innate immune cell output and cytokine production.

**Fig 6:**
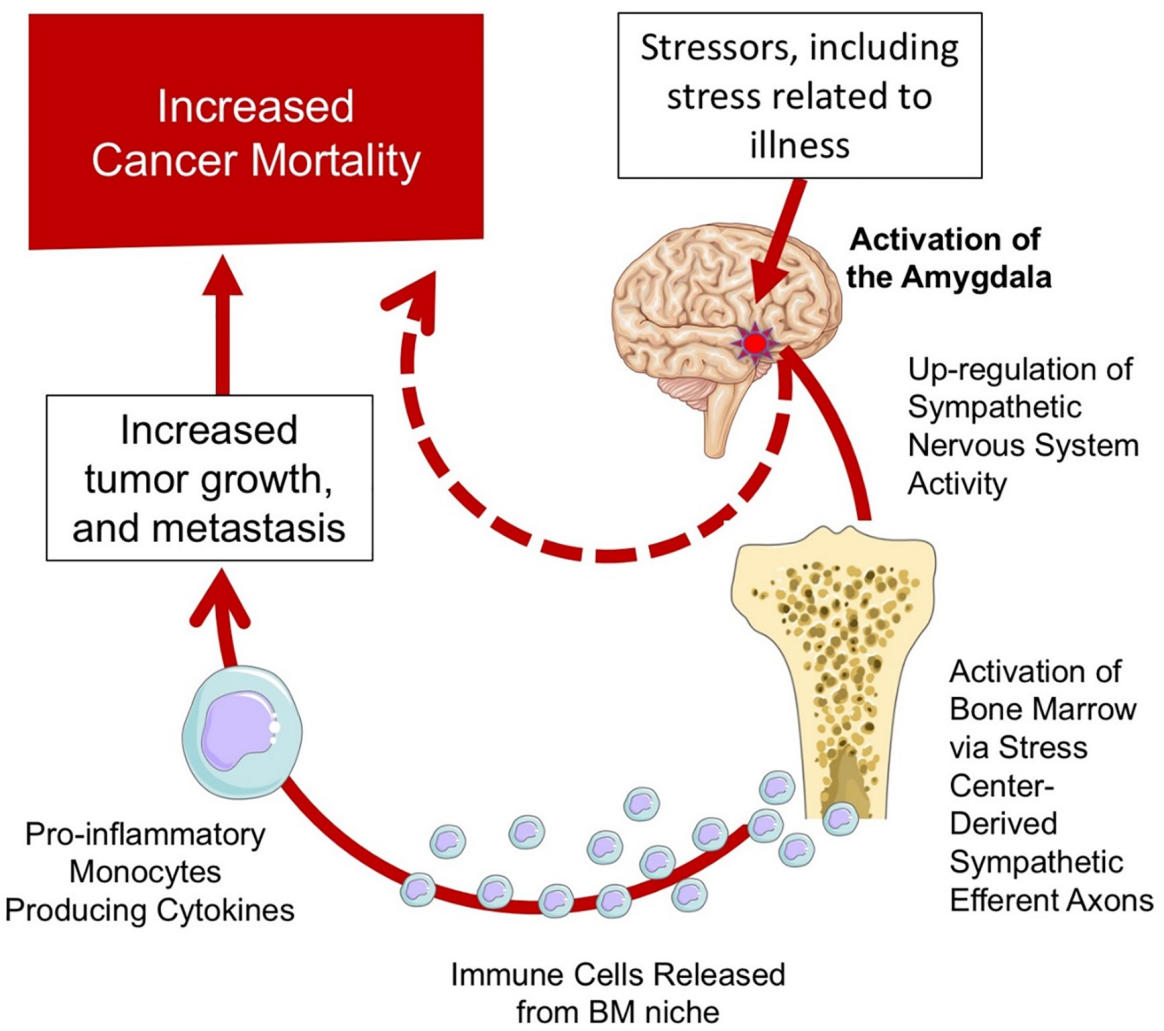
A proposed model of stress leading to worsened cancer survival.

In a recent study, AmygA was found to link to CVD outcomes, in part via up-regulation of bone marrow activity and resultant arterial inflammation [16]. The current findings provide some support for an analogous biological mechanism in the context of cancer. We observed that AmygA associates with heightened hematopoietic tissue activity and leukopoiesis, as well as elevated systemic markers of inflammation. Accordingly, these associative findings raise the hypothesis that an amygdalar-leukopoietic-inflammatory axis may, in part, drive the link between stress, cancer progression, and cancer mortality (Fig 6). Such associations do not prove the existence of a causal pathway; therefore, future studies should test this directly (e.g., through targeted manipulation). Furthermore, future studies should evaluate the directionality of this association (i.e., whether the psychological response to cancer leads to increased inflammation), whether increased inflammation affects the psychological response to cancer, or whether (most likely) the relationship is bidirectional) [39].

The findings from this study suggests an important role for the amygdala in the path between stress and adverse cancer outcomes. Stress, including life stressors and stress related to the oncologic disease, may prompt higher stress-associated neurobiological activity, including increased amygdalar activity. This in turn promotes heightened activity in the sympathetic nervous system, which results in activation of the bone marrow and release of inflammatory cells. This enhanced immune system activity leads to increased tumor growth, metastasis, and worsened outcomes.

Given the association between psychological stress and adverse cancer outcomes, it has been hypothesized that reducing stress may further improve these outcomes. However, studies testing stress reduction approaches among patients with cancer have yielded variable results [40–45]. For example, a pooled analysis of ten randomized stress reduction trials in 1378 cancer patients found improvements in psychological scores without gains in survival [46]. A substantial relative shortcoming of such prior studies is that they were unable to significantly account for inter-individual variability in the stress-response. It is well-appreciated that different individuals could experience markedly different physiologic manifestations of stress to similar stressors [42], which in turn may result in variable responses to stress-reduction interventions. A more objective measure of stress, such as a neurobiological assessment (especially one strongly linked to disease consequences), may enhance the identification of individuals who are most likely to benefit from stress-reducing interventions. Future studies evaluating the impact of stress reduction could specifically target individuals with increased stress-associated neural activity (e.g., heightened AmygA on routine ^18^F-FDG-PET/CT imaging). Further, clinical ^18^F-FDG-PET/CT imaging typically includes the amygdala within the field of view and thus provides the opportunity to measure AmygA, a measure that is reproducible, is stable over several months, and is straight-forward to quantify [13, 47]. Accordingly, AmygA could potentially be measured during staging ^18^F-FDG-PET/CT scans to enhance assessment of prognosis. Future research should evaluate whether measurement of AmygA derived from routine ^18^F-FDG-PET/CT images informs prognosis in oncologic diseases other than HNCA.

Our findings need to be interpreted within the context of the study design. This was a retrospective study among patients with known or suspected HNCA who were being assessed and subsequently treated at a single academic center. However, the population is homogenous, and the association between AmygA and cancer outcomes was robust and remained so even after accounting for cancer- and CVD-specific mortality risk markers. Additionally, we did not measure stress using standardized questionnaires for this cohort. However, in a prior study, we showed a relation between perceived stress and AmygA, thus providing some independent validation of the findings [16]. Further, it is important to note that individuals were aware that they were being evaluated for cancer. This context may have increased anxiety and could have impacted amygdalar activity measured on ^18^F-FDG-PET/CT. It is unclear if AmygA would be similarly predictive of cancer outcomes in other imaging settings.

In conclusion, we observed that resting metabolic amygdalar activity, measured at the time of cancer staging, is a significant predictor of survival among patients with head and neck cancer. Hence, this study provides novel insights into the host-tumor interaction, by illuminating a potential role for a neurobiological mechanism that may substantially alter disease course. Moreover, the study findings provide a rationale for future studies to further investigate and possibly modulate the amygdala-bone marrow-inflammatory axis to improve prognostic assessments, and possibly outcomes, in patients with cancer.

## Supporting information

S1 Table Comparison of baseline variables between those with and without PET imaging. (DOCX)

S2 Table Comparison of cancer variables between those with and without PET imaging. (DOCX)

S3 Table Control tissue activity association with survival. (DOCX)

S4 Table Amygdalar corrected to temporal by tertile activity. (DOCX)

S5 Table Comparison of baseline non-cancer and cancer variables between patients grouped by tertiles of amygdalar activity. (DOCX)

S6 Table Univariate and multivariate analysis of amygdalar activity by tertile vs. mortality in patients with cancer. (DOCX)

S7 Table Threshold for high vs. low amygdalar activity vs. outcomes. (DOCX)

S8 Table Sensitivity analysis of amygdalar activity vs. outcomes. (DOCX)

S1 Fig Kaplan-Meier survival curves of low vs high mean mean amygdalar activity defined based on the median cutoff (A) or the Youden index (B). (DOCX)

S2 Fig Kaplan-Meier survival curves of low vs high mean max amygdalar activity defined based on the median cutoff (A) or the Youden index (B). (DOCX)

S3 Fig Amygdalar activity vs. progression free survival. (DOCX)
