# Supplementary material for: Amygdalar activity measured using FDG-PET/CT at head and neck cancer staging independently predicts survival
Source: PLoS One. 2023 Aug 4;18(8):e0279235. doi: 10.1371/journal.pone.0279235 (PMC10403142; doi:10.1371/journal.pone.0279235)
Supplement: S1 Table — (DOCX) [file pone.0279235.s001.docx]

**Table 1 : Comparison of baseline variables between those with and without PET imaging**

| Variable | All study Cohort  N=1011  (%) | Individuals with Brain Images  N=240  (%) | Individuals without Brain Images  N=771  (%) | *P* Value |
| --- | --- | --- | --- | --- |
| Age (yrs) | **59 (13)** | **60 (14)** | **59 (13)** | **0.55** |
| Female sex, n, (%) | **304 (30.1)** | **73 (30.4)** | **231 (30.0)** | **0.96** |
| Body Mass Index (kg/m^2^) | **27.2 (5.7)** | **27.3 (5.7)** | **27.3 (5.7)** | **0.77** |
| Past Medical History, n, (%) | | | | |
| Reactive airway disease | **125 (12.4)** | **29 (12.1)** | **125 (12.4)** | **0.96** |
| Atrial fibrillation | **42 (4.2)** | **11 (4.6)** | **31 (4.0)** | **0.84** |
| Heart failure | **41 (4.1)** | **7 (2.9)** | **34 (4.4)** | **0.40** |
| Ischemic heart disease | **98 (9.7)** | **20 (8.3)** | **78 (10.1)** | **0.49** |
| Myocardial infarction | **71 (7.0)** | **14 (5.8)** | **57 (7.4)** | **0.49** |
| Stroke | **49 (4.8)** | **8 (3)** | **41 (5.3)** | **0.28** |
| TIA | **25 (2.5)** | **8 (3.3)** | **17 (2.2)** | **0.46** |
| Cardiovascular risk factors, n, (%) | | | | |
| Diabetes | **119 (11.8)** | **26 (10.8)** | **93 (12.1)** | **0.69** |
| Hypertension | **447 (44.2)** | **113 (47.1)** | **334 (43.3)** | **0.34** |
| Dyslipidemia | **278 (27.5)** | **64 (26.7)** | **214 (27.8)** | **0.80** |
| Smoking | **665 (65.8)** | **158 (65.8)** | **507 (65.8)** | **1** |
| Mean ASCVD 10-year risk | **12 (13)** | **12 (13)** | **12 (13)** | **0.99** |
| Laboratory Values | | | | |
| Haematocrit | **38 (5)** | **38 (5)** | **38 (5)** | **0.96** |
| WBC | **8.1 (4.3)** | **7.6 (3.8)** | **8.2 (4.4)** | **0.04** |
| Total Cholesterol (mg/dL) | **171 (31.57)** | **172 (28.4)** | **171 (32.52)** | **0.69** |
| LDL (mg/dL) | **96 (27.81)** | **96 (26)** | **95 (28)** | **0.84** |
| HDL (mg/dL) | **52.8 (14.05)** | **52.4 (13.1)** | **53 (14)** | **0.56** |
| Triglycerides (mg/dL) | **162 (75)** | **166 (72)** | **160 (76)** | **0.37** |
| Glucose (mg/dL) | **116.67 (39)** | **112.3 (39)** | **118.05 (39)** | **0.05** |
| HbA1C | **6.1 (1.2)** | **6 (1.1)** | **6.1 (1.2)** | **0.53** |
| Sodium (mg/dL) | **138 (3)** | **137(3)** | **138 (3)** | **0.08** |
| Creatinine (mg/dL) | **0.94 (0.33)** | **0.93 (0.3)** | **0.94 (0.3)** | **0.63** |
| CRP (mg/dL) | **28.7 (70.78)** | **4.1 (8.2)** | **39.4 (82.5)** | **0.02** |
| Baseline Cardiovascular medications | | | | |
| Statins | **288 (28.5)** | **67 (27.9)** | **221 (28.7)** | **0.88** |
| Beta-blockers | **246 (24.3)** | **59 (24.6)** | **187 (24.3)** | **0.98** |
| Aspirin | **248 (24.5)** | **55 (22.9)** | **193 (25.0)** | **0.56** |
| Angiotensin-converting enzyme inhibitor | **203 (20.1)** | **44 (18.3)** | **159 (20.6)** | **0.49** |
| Angiotensin-receptor blockers | **47 (4.6)** | **16 (6.7)** | **31 (4.0)** | **0.13** |
| Calcium channel blockers | **99 (9.8)** | **17 (7.1)** | **82 (10.6)** | **0.14** |
| Coumadin | **43 (4.3)** | **9 (3.8)** | **9 (3.8)** | **0.79** |
