# Supplementary material for: Amygdalar activity measured using FDG-PET/CT at head and neck cancer staging independently predicts survival
Source: PLoS One. 2023 Aug 4;18(8):e0279235. doi: 10.1371/journal.pone.0279235 (PMC10403142; doi:10.1371/journal.pone.0279235)
Supplement: S2 Table — (DOCX) [file pone.0279235.s002.docx]

**Table 2 : Comparison of cancer variables between those with and without PET imaging**

| Baseline clinical characteristics | All study Cohort  N=1011  (%) | Individuals with Brain Images  N=240  (%) | Individuals without Brain Images  N=771  (%) | P value |
| --- | --- | --- | --- | --- |
| Tumor site | | | | **0.72** |
| Left | **362 (37)** | **83 (35.9)** | **279 (38)** |  |
| Right | **313 (32)** | **75 (32.5)** | **238 (32)** |  |
| Median | **88 (9)** | **25 (10.8)** | **63 (9)** |  |
| Bilateral | **211 (22)** | **48 (20.8)** | **163 (22)** |  |
| Number of radiation cycles, n (%) | | | | **0.40** |
| 1 | **934 (92.4)** | **222 (93)** | **712 (92)** |  |
| 2 | **69 (6.8)** | **15 (6)** | **54 (7)** |  |
| 3 | **6 (0.6)** | **2 (0.8)** | **4 (0.5)** |  |
| 4 | **1 (0.1)** | **1 (0.4)** | **0** |  |
| Type of chemotherapy | | | | |
| Anthracycline | **46 (5)** | **14 (5)** | **32 (4)** | **0.36** |
| Taxol | **415 (41)** | **94 (39)** | **321 (41)** | **0.29** |
| 5 FU | **90 (8)** | **25 (10)** | **65 (8)** | **0.41** |
| Platinum | **643 (63)** | **154 (64)** | **489 (63)** | **0.89** |
| Others | **248 (24)** | **67 (27)** | **181 (23)** | **0.19** |
| Metastases | **214 (21.2)** | **72 (30)** | **142 (18)** | **<0.001** |
