# Supplementary material for: Amygdalar activity measured using FDG-PET/CT at head and neck cancer staging independently predicts survival
Source: PLoS One. 2023 Aug 4;18(8):e0279235. doi: 10.1371/journal.pone.0279235 (PMC10403142; doi:10.1371/journal.pone.0279235)
Supplement: S3 Table — (DOCX) [file pone.0279235.s003.docx]

**Table 3: Control Tissue Activity association with survival**

| Adjustment | | Hazard Ratio from Cox Models | HR for Control Tissue Activity (SUV) Measurement | |
| --- | --- | --- | --- | --- |
|  |  |  | **Temporal lobe** | **Cerebellum** |
| No Co-variates | Univariate | Unstandardized | **0.88 (0.77-1.01)** | **0.89 (0.78-1.02)** |
| Co-variates | **Age and gender** | **Unstandardized** | **0.89 (0.77-1.03)** | **0.89 (0.78-1.03)** |
|  | **ASCVD-score** | **Unstandardized** | **1.01 (0.78-1.03)** | **1.01 (0.99-1.03)** |
|  | **Cancer Stage at baseline** | **Unstandardized** | **0.88 (0.77-1.02)** | **0.89 (0.77-1.02)** |
