# Supplementary material for: Amygdalar activity measured using FDG-PET/CT at head and neck cancer staging independently predicts survival
Source: PLoS One. 2023 Aug 4;18(8):e0279235. doi: 10.1371/journal.pone.0279235 (PMC10403142; doi:10.1371/journal.pone.0279235)
Supplement: S4 Table — (DOCX) [file pone.0279235.s004.docx]

**Table 4: Amygdalar corrected to temporal by tertile activity**

| Variable | Hazard Ratio | 95% Cl  Lower Upper | *P*  Value |
| --- | --- | --- | --- |
| Unadjusted | | | |
| Lower tertile | **Ref (1)** |  |  |
| Middle tertile | **1.59** | **0.83 3.02** | 0.16 |
| Upper tertile | **2.19** | **1.18 4.03** | 0.01 |
| Adjusted for age | | | |
| Lower tertile | **Ref (1)** |  |  |
| Middle tertile | **1.55** | **0.80 2.95** | 0.18 |
| Upper tertile | **2.15** | **1.16 3.9** | 0.01 |
| Adjusted for age and stage | | | |
| Lower tertile | **Ref (1)** |  |  |
| Middle tertile | **1.40** | **0.72 2.71** | 0.31 |
| Upper tertile | 1.92 | 1.02 3.6 | 0.04 |
