# Supplementary material for: Amygdalar activity measured using FDG-PET/CT at head and neck cancer staging independently predicts survival
Source: PLoS One. 2023 Aug 4;18(8):e0279235. doi: 10.1371/journal.pone.0279235 (PMC10403142; doi:10.1371/journal.pone.0279235)
Supplement: S5 Table — (DOCX) [file pone.0279235.s005.docx]

**Supplemental Table 5: Comparison of baseline non-cancer and cancer variables between patients grouped by tertiles of amygdalar activity**

|  | Lower tertile | Middle tertile | Upper tertile | P-value |
| --- | --- | --- | --- | --- |
| Age (yrs) | **59 (15)** | **60 (13)** | **61 (11)** | **0.45** |
| Female sex, n (%) | **24 (30)** | **21 (26)** | **27 (33)** | **0.58** |
| Body Mass Index (kg/m^2^) | **27.5 (4.6)** | **27.1 (5.8)** | **27.1 (6.5)** | **0.90** |
| Cardiovascular risk factors, n (%) | | | | |
| Diabetes | **9 (11)** | **6 (7)** | **11 (13)** | **0.44** |
| Hypertension | **35 (44)** | **34 (42)** | **44 (55)** | **0.23** |
| Dyslipidemia | **16 (20)** | **27 (33)** | **21 (26)** | **0.15** |
| Smoking | **40 (50)** | **36 (45)** | **35 (43)** | **0.70** |
| Mean ASCVD 10-year risk | **12 (15)** | **11 (11)** | **14 (15)** | **0.39** |
| Myocardial infarction | **5 (6)** | **5 (6)** | **4 (5)** | **0.92** |
| Stroke | **1 (1)** | **2 (2)** | **5 (6)** | **0.19** |
| Laboratory Values | | | | |
| Hematocrit | **39 (6)** | **38 (5)** | **37 (5)** | **0.16** |
| Total Cholesterol (mg/dL) | **175 (26)** | **170 (28)** | **171 (30)** | **0.47** |
| LDL (mg/dL) | **97 (25)** | **94 (26)** | **97 (27)** | **0.66** |
| HDL (mg/dL) | **53 (11)** | **52 (15)** | **50 (13)** | **0.34** |
| Triglycerides (mg/dL) | **181 (72)** | **155 (74)** | **162 (69)** | **0.06** |
| Glucose (mg/dL) | **107 (27)** | **106 (22)** | **124 (57)** | **0.004** |
| HbA1C (%) | **5.7 (0.4)** | **5.7 (0.6)** | **6.5 (1.5)** | **0.042** |
| Sodium (mg/dL) | **138 (2.7)** | **138 (3)** | **137 (3.1)** | **0.007** |
| Creatinine (mg/dL) | **0.99 (0.3)** | **0.91 (0.2)** | **0.91 (0.4)** | **0.22** |
| Baseline Cardiovascular medications, n (%) | | | | |
| Statins | **21 (26)** | **24 (30)** | **22 (27)** | **0.88** |
| Beta-blockers | **19 (24)** | **17 (21)** | **23 (28)** | **0.53** |
| Aspirin | **18 (22)** | **17 (21)** | **20 (25)** | **0.85** |
| Angiotensin-converting enzyme inhibitor | **14 (17)** | **14 (17.5)** | **16 (20)** | **0.90** |
| Angiotensin-receptor blockers | **8 (10)** | **5 (6)** | **3 (3)** | **0.26** |
| Calcium channel blockers | **7 (8)** | **3 (3)** | **7 (8)** | **0.35** |
| Coumadin | **3 (3)** | **5 (6)** | **1 (1)** | **0.25** |
| Depression | **9 (11)** | **15 (18)** | **8 (10)** | **0.21** |
| Cancer stage at baseline |  | | | **<0.001** |
| Stage Ι | **2 (2)** | **3 (3)** | **2 (2)** |  |
| Stage ΙΙ | **40 (51)** | **17 (21)** | **11 (13)** |  |
| Stage ΙΙΙ | **7 (9)** | **18 (22)** | **22 (27)** |  |
| Stage IV | **29 (37)** | **42 (52)** | **45 (56)** |  |
| Metastases | **16 (20)** | **27 (33)** | **29 (36)** | **0.06** |
| Detectable lymph node | **60 (76)** | **72 (90)** | **65 (81)** | **0.08** |
| ECOG status, n, (%) | | | | **<0.001** |
| 0 | **56 (70)** | **40 (50)** | **29 (36)** |  |
| 1 | **16 (20)** | **27 (33)** | **31 (38)** |  |
| 2 | **7 (8)** | **13 (16)** | **16 (20)** |  |
| 3 | **0 (0)** | **0 (0)** | **4 (5)** |  |
| Tumor site, n, (%) | | | | |
| Larynx | **8 (10)** | **11 (13)** | **10 (12)** | **0.77** |
| Oropharynx | **31 (39)** | **32 (40)** | **31 (38)** | **0.98** |
| Nasopharynx | **4 (5)** | **10 (12)** | **7 (8)** | **0.25** |
| Hypopharynx | **6 (7)** | **3 (3)** | **3 (3)** | **0.44** |
| Other | **35 (44)** | **26 (33)** | **32 (40)** | **0.33** |
