# Supplementary material for: Amygdalar activity measured using FDG-PET/CT at head and neck cancer staging independently predicts survival
Source: PLoS One. 2023 Aug 4;18(8):e0279235. doi: 10.1371/journal.pone.0279235 (PMC10403142; doi:10.1371/journal.pone.0279235)
Supplement: S6 Table — (DOCX) [file pone.0279235.s006.docx]

**Supplemental Table 6: Univariate and multivariate analysis of amygdalar activity by tertile vs. mortality in patients with cancer**

| Variable | HR (95% CI)  Death | P- value | HR (95% CI)  Death or progression | P-  value |
| --- | --- | --- | --- | --- |
| Unadjusted | | | | |
| Lower tertile | **Ref (1)** |  | **Ref (1)** |  |
| Middle tertile | **1.55 (0.81-2.93)** | **0.17** | **1.58 (0.95-2.62)** | 0.075 |
| Upper tertile | **2.40 (1.29-4.43)** | **0.005** | **2.32 (1.43-3.77)** | <0.001 |
| Adjusted for age | | | | |
| Lower tertile | **Ref (1)** |  | **Ref (1)** |  |
| Middle tertile | **1.51 (0.79-2.86)** | **0.21** | **1.53 (0.92-2.53)** | 0.09 |
| Upper tertile | **2.31 (1.24-4.29)** | **0.008** | **2.24 (1.38-3.63)** | 0.001 |
| Adjusted for ASCVD risk score | | | | |
| Lower tertile | **Ref (1)** |  | **Ref (1)** |  |
| Middle tertile | **1.57 (0.82-2.98)** | **0.17** | **1.64 (0.98-2.74)** | 0.056 |
| Upper tertile | **2.33 (1.25-4.31)** | **0.007** | **2.25 (1.38-3.66)** | 0.001 |
| Adjusted for cancer stage | | | | |
| Lower tertile | **Ref (1)** |  | **Ref (1)** |  |
| Middle tertile | **1.32 (0.68-2.58)** | **0.41** | **1.48 (0.88-2.49)** | 0.14 |
| Upper tertile | 1.96 (1.02-3.76) | 0.04 | 2.10 (1.26-3.49) | 0.004 |
