# Supplementary material for: Amygdalar activity measured using FDG-PET/CT at head and neck cancer staging independently predicts survival
Source: PLoS One. 2023 Aug 4;18(8):e0279235. doi: 10.1371/journal.pone.0279235 (PMC10403142; doi:10.1371/journal.pone.0279235)
Supplement: S7 Table — (DOCX) [file pone.0279235.s007.docx]

**Supplemental Table 7: Threshold for high vs. low amygdalar activity vs. outcomes**

| Variable | HR (95% CI)  Death | P- value | HR (95% CI)  Death or progression | | P-  value |
| --- | --- | --- | --- | --- | --- |
| Primary cut points (Mean mean amygdala) | | | | | |
| Upper tertile vs lower two tertiles | **1.79 (1.11-2.91)** | **0.01** | | **1.83 (1.24-2.68)** | 0.002 |
| Above vs below the median | **1.68 (1.03-2.73)** | **0.04** | | **1.67 (1.14-2.46)** | 0.008 |
| Above vs below Youden index | **2.50 (1.50-4.15)** | **<0.001** | | **1.95 (1.27-3)** | 0.002 |
| Secondary cut points (Mean max amygdala) | | | | | |
| Upper tertile vs lower two tertiles | **2.31 (1.43-3.73)** | **<0.001** | | **1.92 (1.32- 2.81)** | <0.001 |
| Above vs below the median | **1.75 (1.06-2.87)** | **0.02** | | **1.79 (1.21- 2.64)** | 0.003 |
| Above vs below Youden index | 2.48 (1.54-4.01) | <0.001 | | 1.99 (1.35-2.93) | <0.001 |
