# Supplementary material for: Amygdalar activity measured using FDG-PET/CT at head and neck cancer staging independently predicts survival
Source: PLoS One. 2023 Aug 4;18(8):e0279235. doi: 10.1371/journal.pone.0279235 (PMC10403142; doi:10.1371/journal.pone.0279235)
Supplement: S8 Table — (DOCX) [file pone.0279235.s008.docx]

**Supplemental Table 8: Sensitivity analysis of amygdalar activity vs. outcomes**

| Sensitivity analysis | HR (95% Cl) | P-value | HR (95% Cl) | P-value |
| --- | --- | --- | --- | --- |
|  | **Death** | | Death or progression | |
| Uncensored patients | **1.31 (1.05-1.69)** | **0.019** | **1.35 (1.10-1.65)** | 0.003 |
| Adult patients older than 40 year of age | **1.38 (1.08, 1.77)** | **0.01** | **1.35 [1.12, 1.63]** | 0.002 |
| No prior history of cancer | **1.45 (1.04-2.04)** | **0.03** | **1.38 (1.08-1.76)** | 0.009 |
| No prior history of CVD^†^ | **1.35 (1.06-1.70)** | **0.016** | **1.25 (1.03-1.53)** | 0.025 |
| No CV event during follow-up^¶^ | **1.43 (1.10-1.85)** | **0.006** | **1.28 (1.05-1.56)** | 0.012 |
| Patients with advance stage | **1.61 (1.20-2.51)** | **0.001** | **1.35 (1.09-1.67)** | 0.005 |
| Amygdalar activity was measured as mean bilateral amygdalar activity corrected for background cerebral neural tissue activity. ^†^Pre-existing diagnosis of heart failure (HF), coronary artery disease, stroke, myocardial infarction (MI), or transient ischemic attack (TIA) prior to cancer staging. ^¶^Cardiovascular events were defined as ischemic stroke, TIA, MI, or HF. | | | | |
