# Supplementary material for: Amygdalar activity measured using FDG-PET/CT at head and neck cancer staging independently predicts survival
Source: PLoS One. 2023 Aug 4;18(8):e0279235. doi: 10.1371/journal.pone.0279235 (PMC10403142; doi:10.1371/journal.pone.0279235)
Supplement: S1 Fig — (DOCX) [file pone.0279235.s009.docx]

**Supplemental Figure 1: Kaplan-Meier survival curves of low vs high mean mean amygdalar activity defined based on the median cutoff (A) or the Youden index (B)**

**
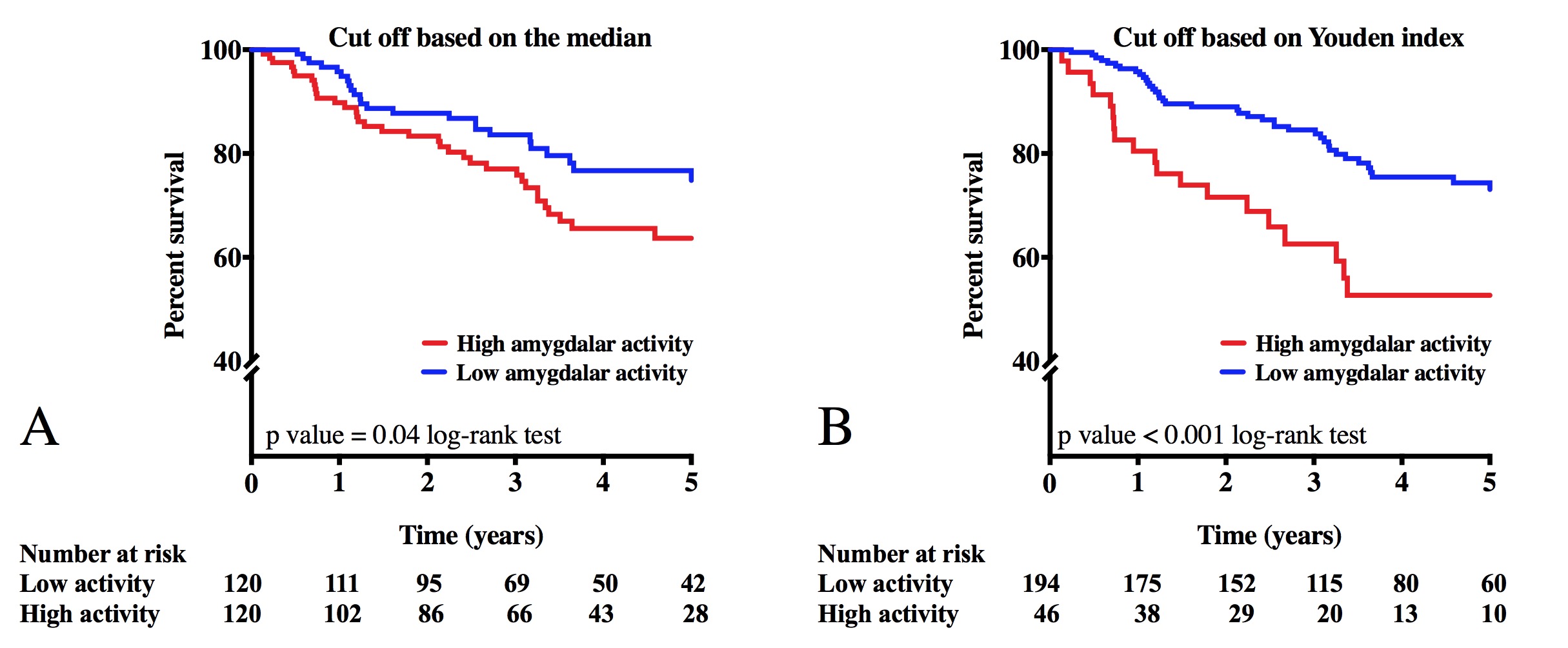
**
