# Supplementary figures and images for: Amygdalar activity measured using FDG-PET/CT at head and neck cancer staging independently predicts survival
Source: PLoS One. 2023 Aug 4;18(8):e0279235. doi: 10.1371/journal.pone.0279235 (PMC10403142; doi:10.1371/journal.pone.0279235)

**Supplemental Figure 3: Amygdalar activity vs. progression free survival**


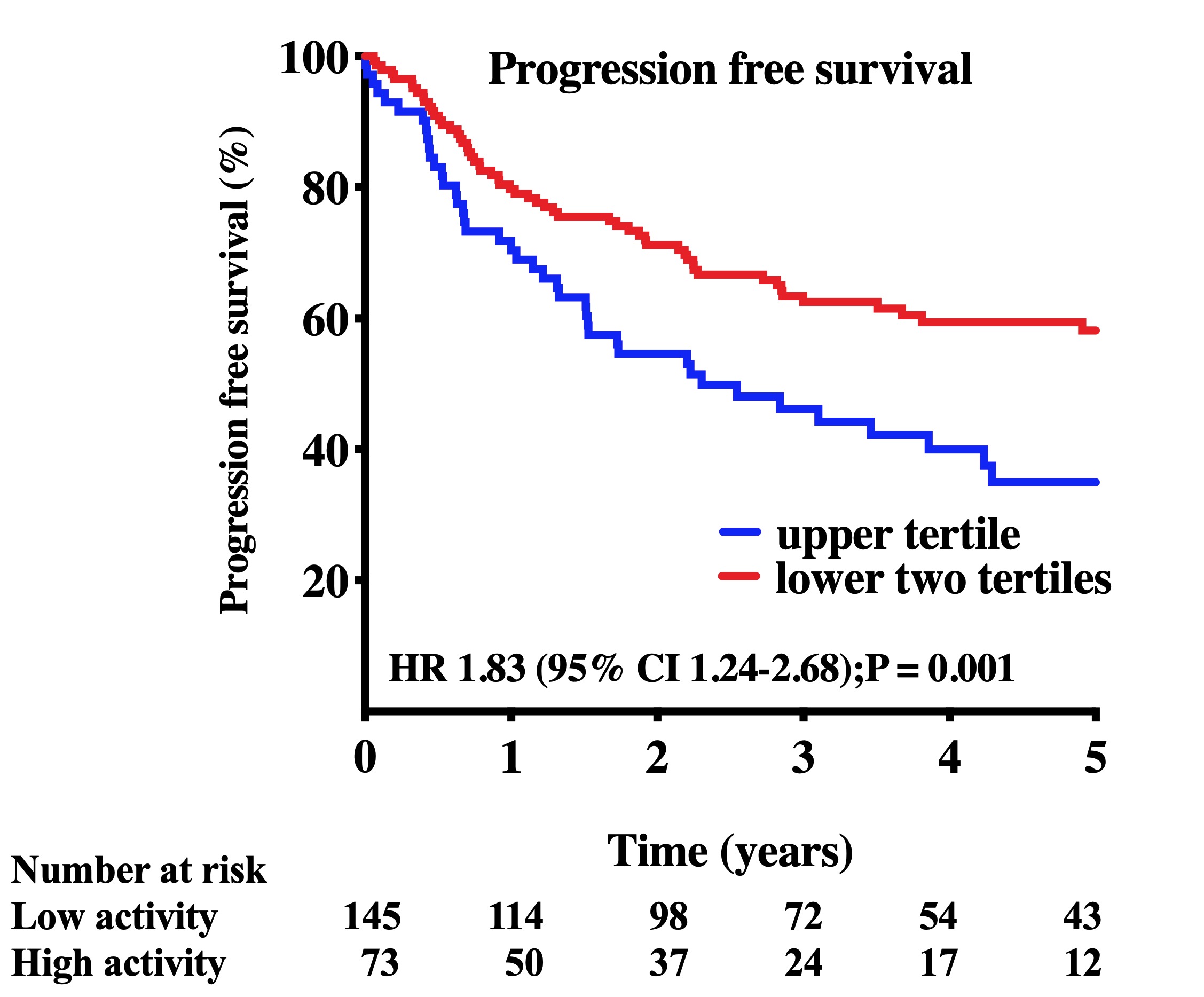

Supplement: S3 Fig — (DOCX) [file pone.0279235.s011.docx]
